# Adverse Effects of Esketamine for the Treatment of Major Depression Disorder: Findings from Randomized Controlled Trials

**DOI:** 10.1007/s11126-020-09871-x

**Published:** 2021-01-07

**Authors:** Siyuan Yang, Jiahe Wang, Xiang Li, Tianyi Wang, Zhongmou Xu, Xiang Xu, Xinmin Zhou, Gang Chen

**Affiliations:** 1 Department of Clinical Medicine, The First Affiliated Hospital of Soochow University, Suzhou, Jiangsu Province China; 2 Department of Clinical Medicine, The Affiliated Jiangyin Hospital, School of Medicine, Southeast University, Jiangyin, Jiangsu Province China

**Keywords:** Esketamine, Major depression disorder, Adverse effects, Meta-analysis

## Abstract

Esketamine is a promising drug which can induce antidepressant effects in Major Depression Disorder (MDD). Several randomized controlled trials (RCTs) have been implemented to assess the efficacy and safety of esketamine for the treatment of MDD. Therefore, we carried out a meta-analysis to assess adverse effect profiles of esketamine for the treatment of MDD. We searched RCTs which were implemented from January 2010 to June 2020 by searching PubMed, Embase and Cochrane Library databases. Finally, four RCTs with 551 patients were included in our study. We pooled 551 patients from 4 RCTs. Compared with placebo, an increased risk of adverse effects was observed in our analysis. After using esketamine, the risk of nausea (RR = 2.34, 95% CI, 1.04 to 5.25, *P* = 0.04), dissociation (RR = 4.54, 95% CI, 2.36 to 8.73, *P* < 0.00001), dizziness (RR = 3.00, 95% CI, 1.80 to 5.00, *P* < 0.0001), vertigo (RR = 7.47, 95% CI, 2.55 to 21.86, *P =* 0.0002), hypoesthesia (RR = 5.68, 95% CI, 2.06 to 15.63, *P* = 0.0008), sedation (RR = 3.96, 95% CI, 1.29 to 12.15, *P* = 0.02) and paresthesia(RR = 3.05, 95% CI, 1.07 to 8.65, *P* = 0.04)were significantly increased compared with placebo. Our synthesized data analysis revealed drug specific risk profiles. The most frequent adverse effects under treatment with esketamine were nausea, dissociation, dizziness, vertigo, hypoesthesia,sedation and paresthesia.

## Introduction

Major depressive disorder (MDD) is a chronic mental disease which may lead to disability and the population of people who suffer from MDD is about 20% of the world population [1]. MDD is also projected to be the main cause of burden of disorder in developed countries by 2030 [2]. MDD is a main reason of morbidity worldwide and is associated with psychosocial and functional impairment, cognitive dysfunction, high risk of suicidal behaviors and excess mortality [3–6]. Major depression disorder is the psychiatric diagnosis most associated with suicide [7]. There are some limitations in currently antidepressant treatment such as delayed onset of efficacy. Approximately one-third of patients with MDD fail to get relief from depression, though they have used multiple biogenic amine antidepressants and thus they get treatment-resistant depression (TRD). Therefore, we need to develop alternate antidepressants which can cause rapid and long-term relief of depressive symptoms to solve the problem of high levels of treatment resistance [8].

Recently, esketamine has been approved as a nasal spray formulation for the treatment of MDD in the American [9]. Proof-of-concept single-dose and repeat-dose studies with intravenous esketamine has shown a significant antidepressant effect and can reduce the risk of suicidal behavior in the short term, with response rates over 60% as early as 4.5 h after a single dose, with a sustained effect after 24 h, and over 40% after 7 days [10]. Esketamine effect can exist continuously over several weeks with repeated doses (two to three doses per week). Intranasal esketamine has shown great efficacy in the treatment of depression and has been granted as a ‘breakthrough therapy’ medicine by US FDA. Theoretically it may offer an improved tolerability profile compared with other antidepressants [11]. Although esketamine has been proved efficient in the treatment of MDD, it also causes some adverse effects. Therefore, we performed a study which included four randomized controlled trials (RCTs) to carry out a comprehensive analysis of adverse effects of the esketamine for the treatment of MDD.

## Methods

### Search Strategy

PubMed, Embase, and Cochrane Library were systematically searched from January 2010 to June 2020 using the following terms: [(“esketamine, major depression disorder,”)] to find the studies we need. Besides, we guaranteed this meta-analysis had included all relevant studies by carefully checking reference documents from studies we chose. We implemented a meta-analysis of data from four published trials according to the PRISMA guidelines.

### Inclusion and Exclusion Criteria

Inclusion criteria indicated as below: (a) Study form: randomized controlled trials; (b) Language limitation: our research had no language limitation; (c) Participants: people who are 18–64 years old with major depression disorder and have no response to at least 1 antidepressant in the depression; (d) Intervention: esketamine and placebo; (e) Outcomes: adverse effects that patients experienced during these studies. Exclusion criteria indicated as below: (a) study forms: case reports, reviews, retrospective studies, protocols and cohort studies; (b) patients as below were excluded: patients with depressive symptoms to esketamine, people who have allergies, hypersensitivity, intolerance or contraindication to esketamine and who have received VNS or DBS, patients who have a current DSM-IV-TR diagnosis of bipolar and related disorders, intellectual disability or cluster b personality disorder, patients who have a current or prior DSM-IV-TR diagnosis of a psychotic disorder, MDD with psychosis, post-traumatic stress disorder (PTSD) or obsessive compulsive disorder (OCD), patients with history of moderate or severe substance or alcohol use disorder.

### Study Selection and Data Collection

All studies that we found in these three databases had been evaluated by inclusion and exclusion criteria that had mentioned. After carefully choosing, the basic information of these studies, the number of patients the study included, and the occurrence side effects were extracted (Table 1).

**Table 1:** characteristic of the included studies and outcome event [7, 8, 10, 12]

Trials	Maggie Fedgchin.2019 NCT02417064	Ella J. Daly.2018 NCT01998958	Carla M Canuso.2018 NCT02133001	Jaskaran B Singh.2016 NCT01640080
**Information of the Included Trials**				
Regions	multicenter in USA	multicenter in USA	multicenter in USA	multicenter in USA
Phases	III	II	III	II
**Elgibility Criteria and Study Design**				
Inclusion Criteria**i**	18–64 years old with recurrent MDD or single-episode MDD (≥2 years), IDS-C30) total score > = 34	20–64 years old with MDD IDS-C30) total score > = 34	18–64 years old with MDD MARDS > = 22 imminent risk of suicide	18–64 years old with MDD
Exclusion Criteria	depressive symptoms to esketamine Received VNS or DBS DSM-5 diagnosis of a psychotic disorder with history of moderate or severe substance or alcohol use disorder	allergies, hypersensitivity, intolerance or contraindication to esketamine/ketamine current clinical diagnosis of bipolar or related disorders intellectual disability, or cluster b personality disorder DSM-5 diagnosis of a psychotic disorder with history of moderate or severe substance Anatomical or medical conditions	current clinical diagnosis of bipolar or related disorders, intellectual disability, or cluster b personality disorder DSM-5 diagnosis of a psychotic disorder with liver or renal insufficiency with uncontrolled hypertension	current clinical diagnosis of bipolar or related disorders intellectual disability, or cluster b personality disorder DSM-5 diagnosis of a psychotic disorder with HIV, hepatitis B, or hepatitis C infection with uncontrolled hypertension had major surgery within 4 weeks before screening
Study Design and The Number of Subjects	esketamine[56 mg]/oral antidepressant esketamine[84 mg]/oral antidepressant oral antidepressant/placebo	esketamine 28 mg esketamine 56 mg esketamine 84 mg	esketamine 84 mg placebo	Intravenous:esketamine 20 mg/kg esketamine .40 mg/kg placebo
**Outcomes Assessments**				
Primary outcomes	Change from Baseline in MADRS Total Score up to Day 28 (MMRM Analysis+ANCOVA Analysis)	Change From Baseline in MADRS Total Score up to Day 8、Day8-Day15(ANCOVA Analysis)	Change from Baseline in MADRS Total Score up to Day 1	Change from Day 1 (baseline) to Day 2 in (MADRS) total score
Safety outcomes	Nausea, dissociation, Dizziness, Vertigo, Headache, Hypoesthesia, Dysgeusia, Vomiting, Anxiety, Somnolence, Euphoric mood, sedation	Nausea, dissociation, Dizziness, Vertigo, Headache, Hypoesthesia, Dysgeusia, Somnolence, Sedation	Nausea, dissociation, Dizziness, Vertigo, Headache, Hypoesthesia, Dysgeusia, Vomiting, Anxiety, Somnolence, Euphoric mood	Nausea, dissociation, Dizziness, Vertigo, Headache, Hypoesthesia, Vomiting

MDD, major depression disorder; VNS, vagal nerve stimulation; DBS deep brain stimulation; DSM the Diagnostic and Statistical Manual of Mental Disorders; MADRS Montgomery-Asberg Depression Rating Scale

### Outcome Measures

Adverse effects that patients experienced included nausea, dissociation, dizziness, vertigo, headache, hypoesthesia, dysgeusia, somnolence, sedation, anxiety, euphoric mood and paresthesia.

### Subgroup Analysis

By analyzing the distinguishing feature of studies included in this meta-analysis, two subgroup analyses were implemented as below: 1. different doses of esketamine; 2. different ways of administration of esketamine. Dosage of intranasal esketamine was divided into 28 mg, 56 mg and 84 mg and dosage of intravenous esketamine was divided into 20 mg/kg and 40 mg/kg. Different ways of administration can be intranasal and intravenous.

### Summary Measures and Synthesis of Results

Review manager 5.3 was used to assess the data. Risk ratios (RRs) with 95%CI based on the random effects model were used to clarify the differences in the incidences of adverse effects. The statistical heterogeneity was evaluated by the I^2^ statistic as below: the heterogeneity of I^2^ < 30% is low, the heterogeneity of 30% < I^2^ < 50% is moderate and the heterogeneity of I^2^ > 50% is substantial. The stability of the consolidated results is evaluated by subgroup analysis. A < 0.05 *P* value was significant for all analyses.

### Risk of Bias

We used the Review Manager 5.3 software to explore the risk of bias in these studies included. The Cochrane collaboration uniform criteria were used to assess the risk of bias of RCTs.

## Results

### Search Results

**Fig. 1:**
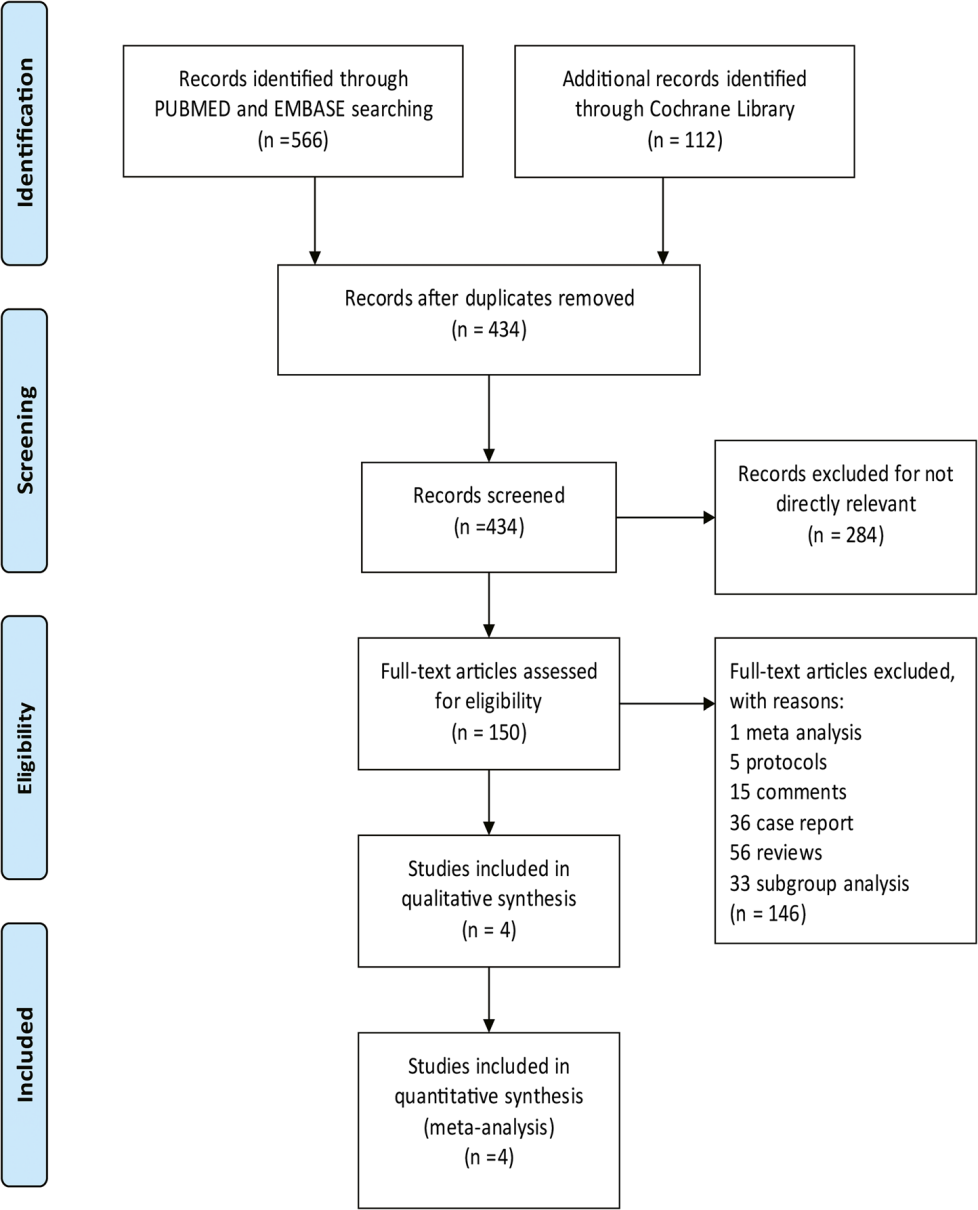
The study search, selection and inclusion process

Five hundred sixty-six articles from PubMed and Embase, and 112 from Cochrane library were identified. 434 articles were left after articles that duplicates were removed, and 284 articles which were not relevant to the subject were removed. Thus, 150 of these articles related to the topic of interest were left. However, among them, 113 articles were excluded because they were protocols, case report, reviews and comments. Besides, 33 articles on subgroup analysis of RCTs were excluded. Therefore, finally, we included these 4 RCTs in our study (Fig. 1).

### Assessment of Adverse Events

**Fig. 2:**
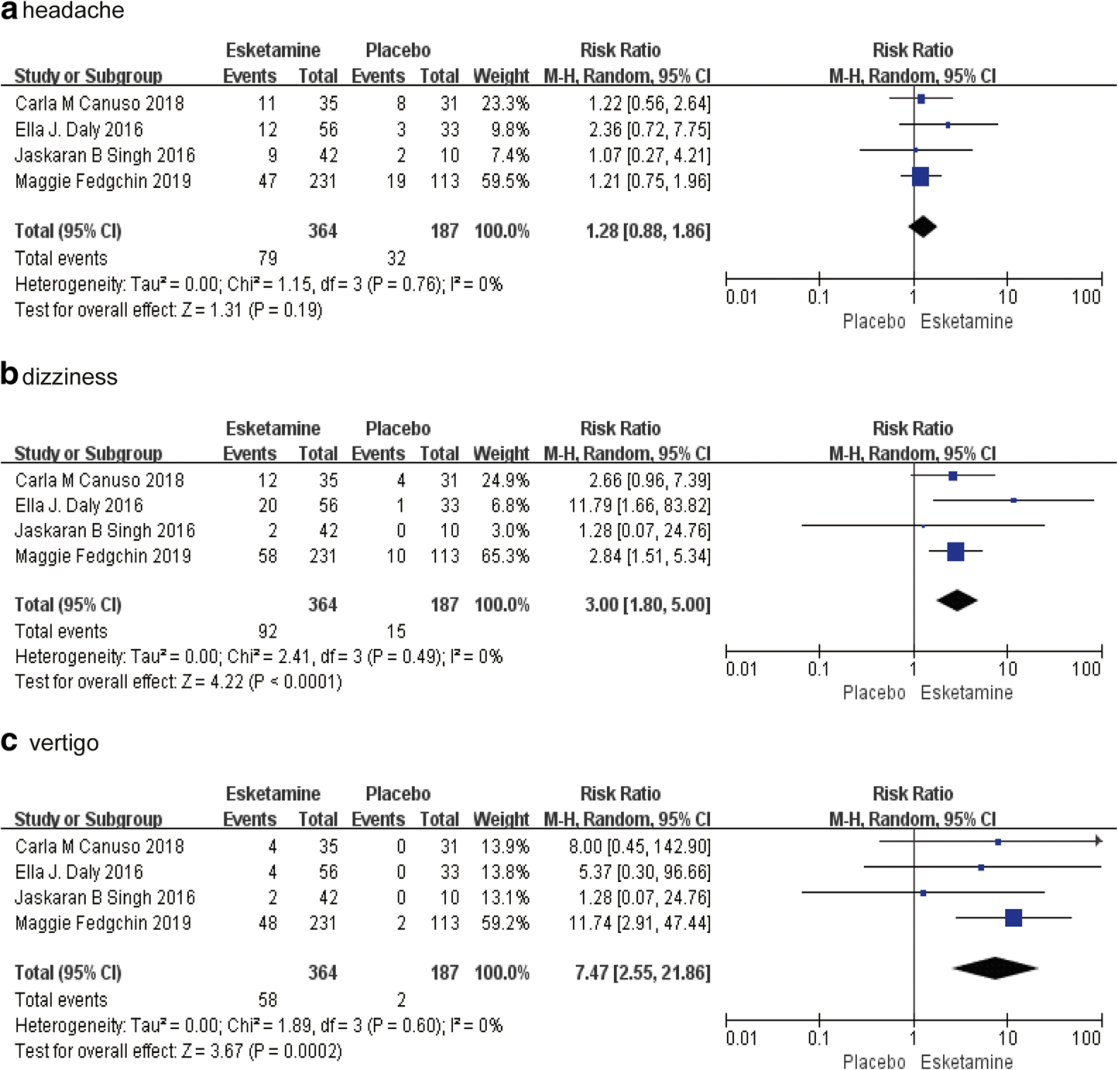
The pooled RR of head discomfort. The blue square indicates the estimated RR for each RCT. The size of blue square indicates the estimated weight of each RCT, and the extending lines indicate the estimated 95% CI of RR for each RCT. The black diamond indicates the estimated RR (95% CI) for all patients together. (**a**) Headache (**b**) Dizziness (**c**) Vertigo. CI, confidence interval; RCT, randomized controlled trial; RR: risk ratio

**Fig. 3:**
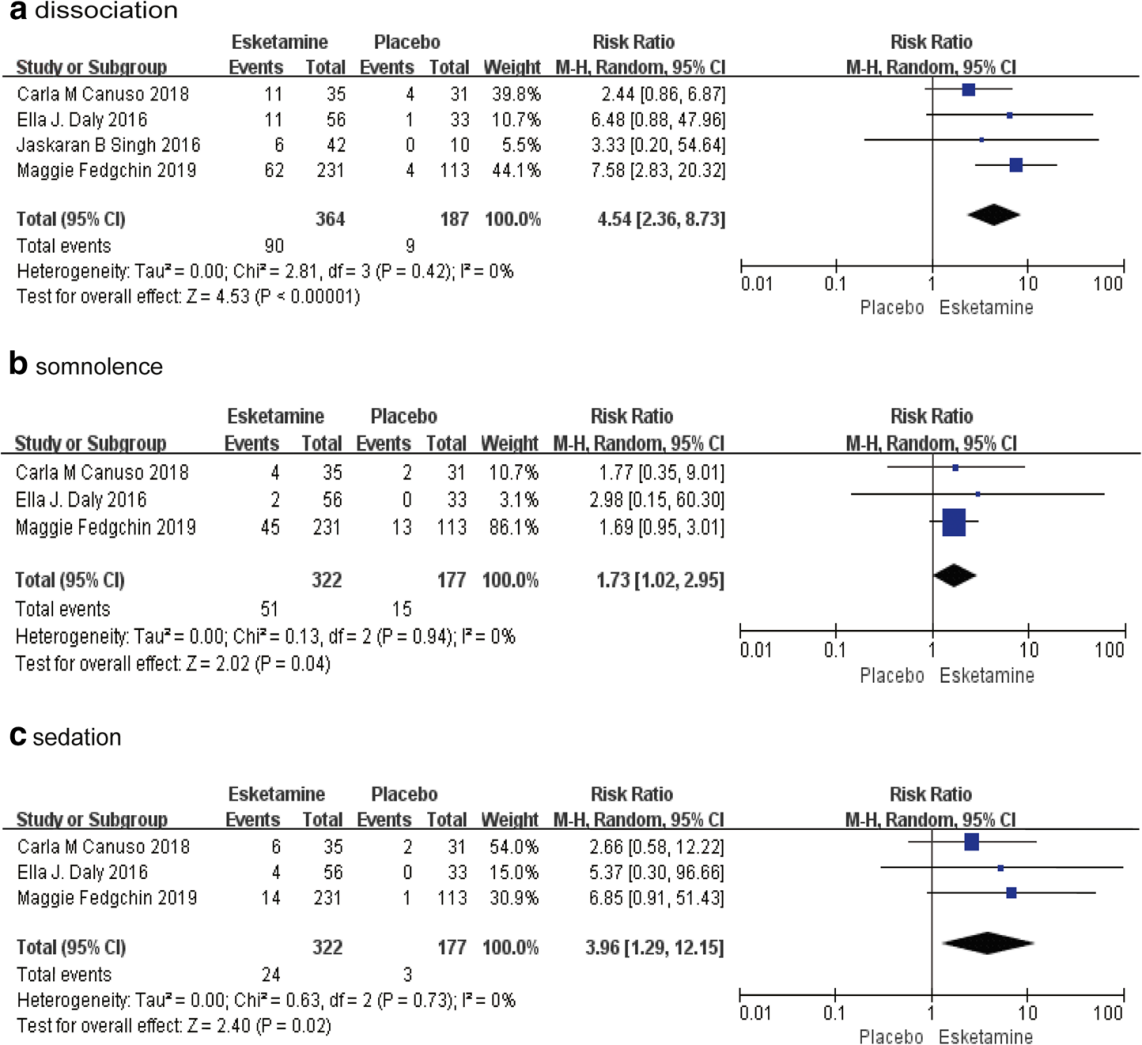
The pooled RR of psychiatric symptoms. The blue square indicates the estimated RR for each RCT. The size of blue square indicates the estimated weight of each RCT, and the extending lines indicate the estimated 95% CI of RR for each RCT. The black diamond indicates the estimated RR (95% CI) for all patients together. (**a**) Dissociation (**b**) Somnolence (**c**) Sedation. CI, confidence interval; RCT, randomized controlled trial; RR: risk ratio

**Fig. 4:**
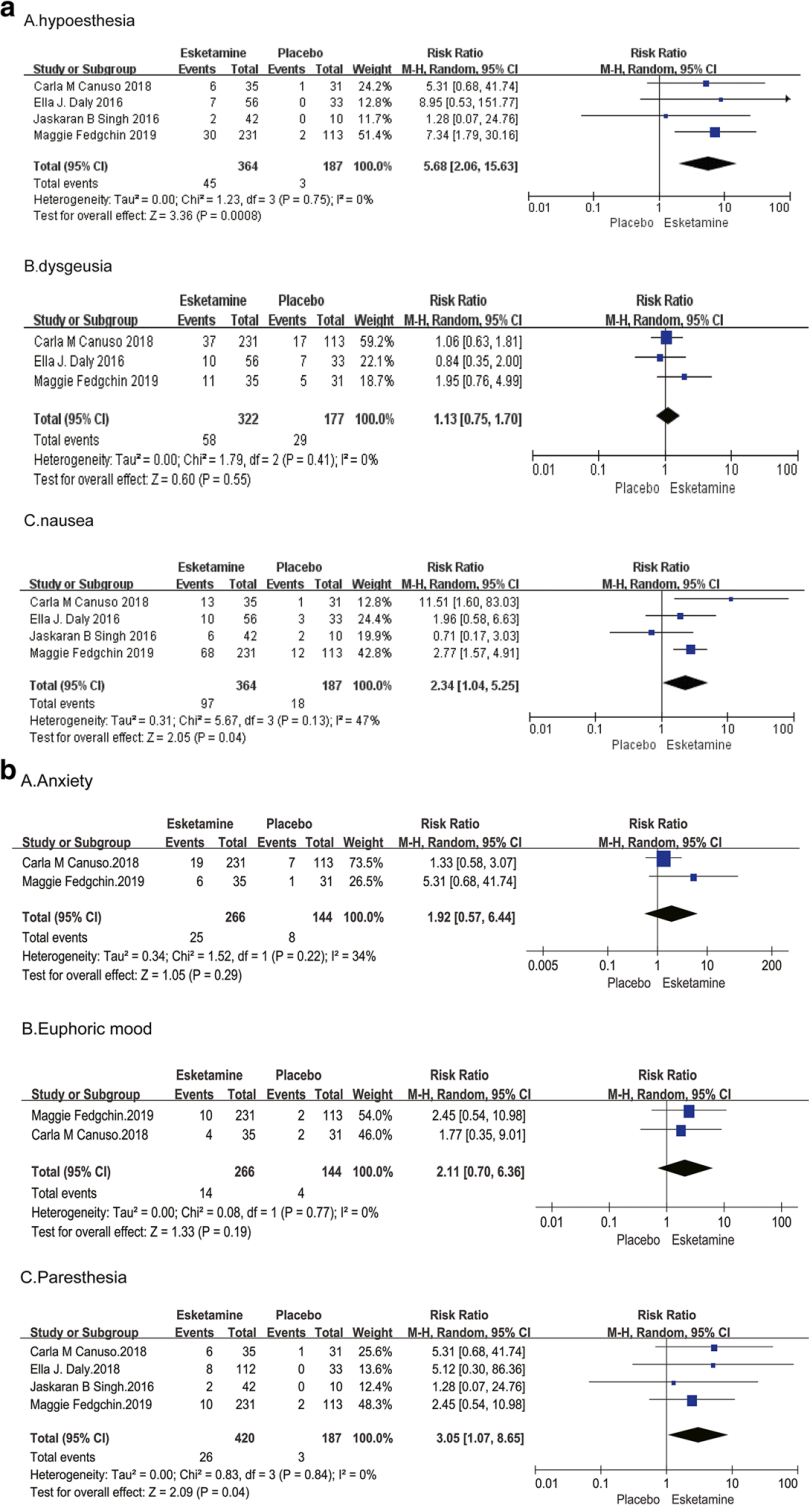
(**a**) The pooled RR of adverse events of sensory system symptoms. The blue square indicates the estimated RR for each RCT. The size of blue square indicates the estimated weight of each RCT, and the extending lines indicate the estimated 95% CI of RR for each RCT. The black diamond indicates the estimated RR (95% CI) for all patients together. (A) Dysgeusia (B) Hypoesthesia (**c**) Nausea. CI, confidence interval; RCT, randomized controlled trial; RR: risk ratio. (**b**) The pooled RR of adverse events of sensory system symptoms. The blue square indicates the estimated RR for each RCT. The size of blue square indicates the estimated weight of each RCT, and the extending lines indicate the estimated 95% CI of RR for each RCT. The black diamond indicates the estimated RR (95% CI) for all patients together. (A) Anxiety (B) Euphoric mood (C) Paresthesia. CI, confidence interval; RCT, randomized controlled trial; RR: risk ratio

We analyzed several adverse events, including nausea, dissociation, dizziness, vertigo, headache, hypoesthesia, dysgeusia, somnolence and sedation. We divided these adverse effects into three parts (head discomfort, psychiatric symptoms and other symptoms) to display. In esketamine group, the incidence of nausea, dissociation, dizziness, vertigo, hypoesthesia, somnolence or sedation is higher than the placebo group, while the proportion of headache and dysgeusia have no significant difference (head discomfort: dizziness: RR = 3.00, 95% CI, 1.80 to 5.00, *P* < 0.0001; vertigo: RR = 7.47, 95% CI, 2.55 to 21.86, *P =* 0.0002; headache: RR = 1.28, 95% CI, 0.88 to 1.86, *P* = 0.19 (Fig. 2), psychiatric symptoms: dissociation: RR = 4.54, 95% CI, 2.36 to 8.73, *P* < 0.00001; somnolence: RR = 1.73, 95% CI, 1.02 to 2.95, *P* = 0.04; sedation: RR = 3.96, 95% CI, 1.29 to 12.15, *P* = 0.02 (Fig. 3), other symptoms: hypoesthesia: RR = 5.68, 95% CI, 2.06 to 15.63, *P* = 0.0008; dysgeusia: RR = 1.13, 95% CI, 0.75 to 1.70, *P* = 0.55; nausea: RR = 2.34, 95% CI, 1.04 to 5.25, *P* = 0.04; (Fig. 4a)anxiety:RR = 1.94, 95%CI, 0.57 to 6.44, *P* = 0.22; euphoric mood: RR = 2.11, 95% CI, 0.70 to 6.36, *P* = 0.77; paresthesia: RR = 3.05, 95% CI, 1.07 to 8.65, *P* = 0.84; (Fig. 4b).

### Subgroup Analysis

By analyzed previous clinical trials we chose, the dosage of intranasal esketamine were roughly divided into 28 mg, 56 mg and 84 mg. In 28 mg subgroup, it has no significant difference in adverse effects listed in the article (nausea: *P* = 0.87, dissociation: *P* = 0.30, dizziness: *P* = 0.07, vertigo: *P* = 0.16, headache: *P* = 0.05, somnolence: *P* = 0.16, sedation: *P* = 0.31, hypoesthesia: *P* = 0.31, dysgeusia: *P* = 0.35, Paresthesia: *P* = 0.15). In 56 mg and 84 mg subgroup, the proportion of patients who have nausea (56 mg: RR = 2.48, 95% CI, 1.41 to 4.35, *P* = 0.002; 84 mg: RR = 3.23, 95% CI, 1.91 to 5.49, *P* < 0.0001), dissociation (56 mg: RR = 8.06, 95% CI, 3.27 to 19.91, *P* < 0.00001; 84 mg: RR = 4.77, 95% CI, 2.03 to 11.23, *P* = 0.0003), dizziness (56 mg: RR = 4.69, 95% CI, 1.31 to 16.73, *P* = 0.02; 84 mg: RR = 3.21, 95% CI, 1.53 to 6.75, *P* = 0.002), vertigo (56 mg: RR = 10.16, 95% CI, 2.78 to 37.04, *P* = 0.0004; 84 mg: RR = 9.91, 95% CI, 3.04 to 32.26, *P* = 0.0001), hypoesthesia (56 mg: RR = 8.0, 95% CI, 2.19 to 29.45, *P* = 0.002; 84 mg: RR = 7.17, 95% CI, 2.39 to 21.56, *P* = 0.0005), sedation (56 mg: RR = 6.55, 95% CI, 1.17 to 36.51, *P* = 0.03; 84 mg: RR = 4.09, 95% CI, 1.30, 36.51, *P* = 0.02) or paresthesia(56 mg: RR = 7.00, 95% CI, 2.18 to 22.43, *P* = 0.001; 84 mg: RR = 4.07, 95% CI, 1.21, 13.70, *P* = 0.02) in esketamine group is more than the placebo group. From these data, we found the most common adverse effects under treatment with esketamine were vertigo, dissociation, hypoesthesia, dizziness and nausea. However, in 56 mg and 84 mg subgroup, it has no significant difference in headache, dysgeusia and somnolence (headache: 56 mg: RR = 1.24, 95% CI, 0.74 to 2.07, *P* = 0.42; 84 mg: RR = 1.27, 95% CI, 0.83 to 1.95, *P* = 0.27, dysgeusia: 56 mg: RR = 0.92, 95% CI, 0.53 to 1.60, *P* = 0.77; 84 mg: RR = 1.34, 95% CI, 0.86 to 2.10, *P* = 0.20, somnolence: 56 mg: RR = 1.81, 95% CI, 0.97 to 3.38, *P* = 0.06; 84 mg: RR = 1.60, 95% CI, 0.88 to 2.90, *P* = 0.12, anxiety: 56 mg: RR = 1.44, 95% CI, 0.53 to 3.93, *P* = 0.47; 84 mg: RR = 1.86, 95% CI, 0.76 to 4.52, *P* = 0.19, euphoric mood: 56 mg: RR = 4.15, 95% CI, 0.86 to 19.99, *P* = 0.98; 84 mg: RR = 0.97, 95% CI, 0.13 to 7.03, *P* = 0.12).

The administration of esketamine included two ways: intranasal and intravenous. Intravenous esketamine has no significant difference in safety compared with the placebo group (nausea: RR = 0.71, 95% CI, 0.17 to 3.03, *P* = 0.65, dissociation: RR = 3.33, 95% CI, 0.20 to 54.64, *P* = 0.40, dizziness: RR = 1.28, 95% CI, 0.07 to 24.76, *P* = 0.87, vertigo: RR = 1.28, 95% CI, 0.07 to 24.76, *P* = 0.87, headache: RR = 1.07, 95%CI, 0.27 to 4.21, *P* = 0.92, hypoesthesia: RR = 1.28, 95% CI, 0.07 to 24.76, *P* = 0.87, paresthesia: RR = 1.30, 95%CI, 0.06 to 29.10, *P* = 0.87). In the intranasal esketamine, the proportion of people who have nausea, dissociation, dizziness, vertigo or hypoesthesia in esketamine group is more than the placebo group. However, the proportion of people who have headache and paresthesia (RR = 1.30, 95%CI, 0.88,1.92, *P* = 0.18) in esketamine group has no significant difference (Table 2).

**Table 2:** Subgroup analyses of safety outcome

Safety																		
	Nausea		Dissociation		Dizziness		Vertigo		Headache		Hypoesthesia		Dysgeusia		Somnolence		sedation	
	RR, 95%CI	*P* value	RR, 95%CI	P value	RR, 95%CI	P value	RR, 95%CI	P value	RR, 95%CI	P value	RR, 95%CI	P value	RR, 95%CI	P value	RR, 95%CI	P value	RR, 95%CI	P value
Dose(intranasal)																		
28 mg	1.16 [0.21, 6.32]	0.87	3.47 [0.34,35.82]	0.30	6.95 [0.84, 57.73]	0.07	8.50 [0.43, 168.30]	0.16	3.47 [0.98, 12.32]	0.05	5.10 [0.22, 119.32]	0.31	0.50 [0.11,2.15]	0.35	8.50 [0.43, 168.30]	0.16	5.10 [0.22,119.32]	0.31
56 mg	2.48 [1.41, 4.35]	0.002	8.06 [3.27, 19.91]	<0.00001	4.69 [1.31, 6.73]	0.02	10.16 [2.78, 37.04]	0.0004	1.24 [0.74, 2.07]	0.42	8.02 [2.19, 29.45]	0.002	0.92 [0.53,1.60]	0.77	1.81 [0.97, 3.38]	0.06	6.55 [1.17, 36.51]	0.03
84 mg	3.23 [1.91, 5.49]	<0.0001	4.77 [2.03, 11.23]	0.0003	3.21 [1.53, 6.75]	0.002	9.91 [3.04, 32.26]	0.0001	1.27 [0.83, 1.95]	0.27	7.17 [2.39, 21.56]	0.0005	1.34 [0.86,2.10]	0.20	1.60 [0.88, 2.90]	0.12	4.09 [1.30, 36.51]	0.02
Subgroup Difference	p = 0.47		*p* = 0.64		*p* = 0.74		*p* = 0.99		p = 0.31		*p* = 0.97		*p* = 0.32		*p* = 0.56		*p* = 0.90	
Intranasal	2.93 [1.58, 5.45]	0.0007	4.64 [2.03, 10.63]	0.0003	3.10 [1.81, 5.32]	<0.0001	9.75 [3.08, 30.87]	0.001	1.30 [0.88, 1.92]	0.18	6.91 [2.35, 20.32]	0.0004	N/A	N/A	N/A	N/A	N/A	N/A
Intravenous	3.33 [0.20, 54.64]	0.40	3.33 [0.20, 54.64]	0.40	1.28 [0.07, 24.76]	0.87	1.28 [0.07, 24.76]	0.87	1.07 [0.27, 4.21]	0.92	1 .28 [0.07, 24.76]	0.87	N/A	N/A	N/A	N/A	N/A	N/A
Subgroup Difference	*p* = 0.08		*p* = 0.82		p = 0.56		*p* = 0.21		*p* = 0.79		*p* = 0.29		N/A		N/A		N/A	
Safety																		
	Anxiety		Euphoric mood		Paresthesia													
	RR, 95%CI	P value	RR, 95%CI	P value	RR, 95%CI	P value												
Dose(intranasal)																		
28 mg	N/A	N/A	N/A	N/A	9.57 [0.44, 210.54]	0.15												
56 mg	1.44 [0.53, 3.93]	0.47	4.15 [0.86, 19.99]	0.98	7.00 [2.18, 22.43]	0.001												
84 mg	1.86 [0.76, 4.52]	0.19	0.97 [0.13, 7.03]	0.08	4.07 [1.21, 13.70]	0.02												
Subgroup Difference	*p* = 0.71		p = 0.12		P < 0.0001													
Intranasal	N/A	N/A	N/A	N/A	2.90 [0.72, 11.75]	0.13												
Intravenous	N/A	N/A	N/A	N/A	1.30 [0.06, 29.10]	0.87												
Subgroup Difference	p = 0.08		p = 0.82		p = 0.56													

### Risk of Bias in Included Studies

**Fig. 5:**
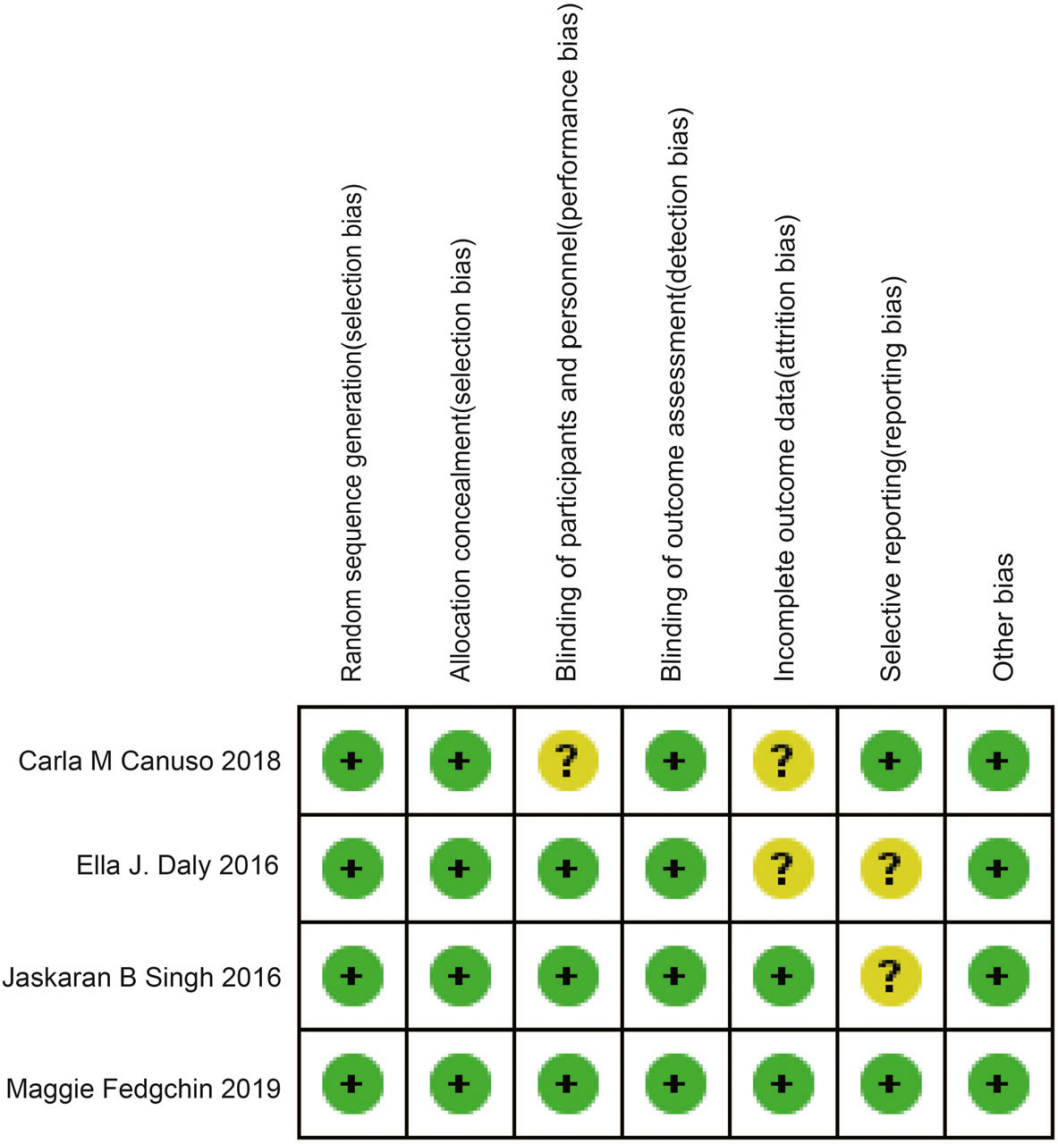
Risk of bias: a summary table for each risk of bias item for each study

The risk of bias of the four included RCTs is shown in Fig. 5. The risk for performance bias is unclear in Carla’s study in 2018. The risk for attrition bias is unclear in Carle’s study in 2018 and Ella’s study in 2016. The risk for reporting bias is unclear in Ella’s study in 2016 and Jaskaran’s study in 2016.

## Discussion

Our study included 551 patients from 4 RCTs which supplied high levels of clinical reliability to our study to assess the adverse effects of esketamine for the treatment of Major Depression Disorder [12]. In recent years, some studies had comprehensively described the adverse effects of esketamine. The study performed by Ella had reported the adverse effects that occurred in more than 10% of all the patients included dizziness (39%), dysgeusia (23%), nausea (16%), headache (14%) and sedation (11%). However, adverse events were inconsistent in these RCTs. After our study, we found that the most common adverse effects under treatment with esketamine were vertigo, hypoesthesia, dissociation, sedation, dizziness, nausea and paresthesia followed by headache, somnolence, dysgeusia, anxiety and euphoric mood. During subgroup analysis, we found the incidence of adverse events showed no statistical significance in patients whose dosage of esketamine was 28 mg. In 56 mg and 84 mg subgroup, there was a significantly increase in the incidence of adverse events such as dissociation, vertigo, dizziness, nausea, hypoesthesia, sedation and paresthesia compared with the placebo group. We also found the incidence of adverse events in 56 mg and 84 mg showed no significant difference except nausea, dissociation, dizziness, vertigo, hypoesthesia, sedation and paresthesia. For this reason, dose-related safety events were not found except nausea,dissociation, dizziness, vertigo, hypoesthesia, sedation and paresthesia. However, these RCTs assessed only the short-term adverse events of esketamine, we evaluated adverse events in these studies only in 4 weeks after the first evaluations of these studies on adverse events were limited to 4 weeks after the first dose. Therefore, whether esketamine will produce long-term adverse events or not still needs further observation and verification. Thus, we should pay attention to these short-term adverse events especially vertigo, dissociation, hypoesthesia, sedation, nausea, dizziness and paresthesia in the process of treatment for MDD with esketamine. We also found most adverse events occurring on dosing days were transient and either mild or moderate in severity, so we can pay more attention to adverse events on dosing days, but we also should care these adverse events after dosing days.

We also compared the incidence of adverse events of different ways of administration of esketamine: intranasal and intravenous. Intravenous studies were dosed by weight whereas intranasal studies were not. In the intranasal esketamine subgroup, we found intranasal esketamine mostly caused nausea, dissociation, dizziness, vertigo and hypoesthesia, However, due to the number of RCTs is not enough, the data we analyzed was not effective, we could not draw a clear conclusion in the intravenous esketamine subgroup, but it would be worthwhile for future studies to shed light on the actual difference in safety between intranasal and intravenous esketamine. What is more, a study has been designed to investigate repeated administration of oral esketamine in patients with TRD. Therefore, we can study the advantages of different ways of treatment strategies of MDD in the future [13].

Our study also has a few limitations. Although patients in these studies used antidepressants before, all these studies we selected lacked the comparison of esketamine with other therapeutic medications. In the future, more studies are needed to compare whether esketamine is more effective than other therapeutic medications. When assessing difference of adverse effect in different ways of administration of esketamine, there is not enough study in intravenous esketamine.The single small intravenous esketamine double-blind placebo-controlled study is obviously too small to comment reliably on any differences from the intranasal studies. This is a very small study adds little to the data and its inclusion is questionable. Therefore, the accuracy of the results needs further verification. Thus, more studies are needed for assessing the safety, efficacy and tolerability of intranasal esketamine and intravenous esketamine. Moreover, the small sample size and enrollment criteria that excluded individuals with a history of psychotic symptoms and other situations limit some study findings. Therefore, in order to prove that the extensive application of esketamine on different kinds of people, more studies on MDD patients of different characteristics are still needed.

## Conclusions

Because of the meta-analytic comparison between esketamine and placebo, our study revealed drug specific risk profiles and incidence of adverse effects in different doses of esketamine. The most frequent adverse effects under treatment with esketamine were nausea, dissociation, dizziness, vertigo, hypoesthesia, sedation and paresthesia. Therefore, when determining the administration of esketamine, potential adverse effects should be considered.
